# Supplementary material for: Dynamic transcriptomic profiles of zebrafish gills in response to zinc supplementation
Source: BMC Genomics. 2010 Oct 11;11:553. doi: 10.1186/1471-2164-11-553 (PMC3091702; doi:10.1186/1471-2164-11-553)
Supplement: Additional file 2 — Interactive Direct Interaction Network representing the molecular interactions between zinc, copper, iron, calcium and proteins encoded by transcripts changed by zinc supplementation. Mini web-site containing index.html and hyperlinked pages in subdirectory describing a Direct Interaction Network automatically generated based on curated interactions contained within the proprietary PathwayArchitect database. Ovals represent proteins and the circles symbolize metal ions. Objects are coloured by their abundance in zebrafish at the time-point they were significantly different from the control is a scale from -4 fold (dark green) to +4 fold (dark red). Where significant differences were found at more than one time-point, the colour overlay shows expression at the first instance. Dark blue squares denote 'binding', and light blue squares 'expression'; green squares stand for 'regulation', green diamonds for 'metabolism', and green circles for 'promoter binding'. Arrow heads indicate directionality of the interaction where annotated. All nodes and edges can be further interrogated by selecting the relative area of the image. [file 1471-2164-11-553-S2.zip › PathwayArchitect Zn xs DIN/1494190.html]

# REGULATION:

|  |  |
| --- | --- |
| Type | REGULATION |
| Effect | None |


---

|  |  |
| --- | --- |
| Score | 0 |


---

|  |  |
| --- | --- |
| Reference Count | 6 |


---

|  |  |
| --- | --- |
| Mechanism | Unknown |


---

|  |  |
| --- | --- |
| Reference:0 || Sentence | "This is influenced by Cu binding compounds, sulphide production in the rumen, and molybdenum and zinc concentrations of the diet." |
| PMID | 6388137 |
| Year | 1984 |
| Species | Rat |
|  | Mouse |
| Journal | Vet Q |
| RefScore | 0 |
| Source | PArchNLP |
  |
|  |  |


---

|  |  |
| --- | --- |
 Reference:1 || Sentence | Copper (Cu) deficiency decreases the activity of Cu-dependent antioxidant enzymes such as Cu,zinc-superoxide dismutase (Cu,Zn-SOD) and may be associated with increased susceptibility to oxidative stress. |
| Year | 2005 |
| PMID | 16098731 |
| Species | Rat |
| Journal | J Nutr Biochem |
| RefScore | 1 |
| Source | PArchNLP |
  ||  |  |


---

|  |  |
| --- | --- |
 Reference:2 || Sentence | Batch adsorption experiments indicated that the activated sludge adsorption capacity was highest for Cu, followed by Cd, Ni, and then Zn. |
| Year | 2004 |
| PMID | 14675654 |
| Journal | Water Res |
| RefScore | 2 |
| Source | PArchNLP |
  ||  |  |


---

|  |  |
| --- | --- |
 Reference:3 || Sentence | Cr or Cu depletion significantly affected iron, zinc, and magnesium concentrations in the liver. |
| Year | 2004 |
| PMID | 14990268 |
| Species | Rat |
| Journal | Nutrition |
| RefScore | 1 |
| Source | PArchNLP |
  ||  |  |


---

|  |  |
| --- | --- |
 Reference:4 || Sentence | Averaged values of the metals for the three categories revealed that Zn is the most abundant metal, followed by Fe, then Mn and Cu the least. |
| PMID | 15745247 |
| Year | 2004 |
| Species | Human |
| Journal | Biomed Environ Sci |
| RefScore | 1 |
| Source | PArchNLP |
  ||  |  |


---

|  |  |
| --- | --- |
 Reference:5 || Sentence | There was a correlation between BMD with the Ca, Zn of the hair and dietary Ca, Zn, Cu or between dietary Zn with Ca, Zn in the hair and Ca, Cu intakes. |
| PMID | 15934321 |
| Year | 2005 |
| Species | Human |
| Journal | J Huazhong Univ Sci Technolog Med Sci |
| RefScore | 2 |
| Source | PArchNLP |
  |


---

|  |  |
| --- | --- |
